# Dietary polyphenols link extracellular histones and nonhistone proteins

**DOI:** 10.1016/j.jbc.2022.102651

**Published:** 2022-10-27

**Authors:** Jiuyang Liu, Tatiana G. Kutateladze

**Affiliations:** Department of Pharmacology, University of Colorado School of Medicine, Aurora, Colorado, USA

**Keywords:** histone, polyphenol, protein, antioxidant, diet, EGCG, (-)-epigallocatechin-3-O-gallate

## Abstract

Numerous studies have demonstrated antioxidant, anti-inflammatory, antimicrobial, anticancer, and cardio-protective activities of dietary polyphenols, but due to diverse structures and subclasses of polyphenols, little is known about their mechanisms of action. The study by Yamaguchi *et al.* published in JBC provides mechanistic insights into how dietary polyphenols confer histone-binding ability on certain proteins and motivates the research community to further explore health benefits of polyphenols.

Polyphenols constitute a large family of organic compounds containing one or more hydroxylated aromatic rings. Produced by plants as metabolites, polyphenols are abundantly present in fruit, vegetables, tea, herbs, and wine, essentially accounting for their flavor and color and often for their pharmacological properties. Polyphenols have been extensively studied as natural therapeutic tools beneficial in preventing various human diseases and ailments, but they have also been found to impact disease progression and even exhibit curative properties (1, 2). Despite recent interest in exploring the therapeutic potential of polyphenols, especially in combination with existing drugs and conventional therapies, our understanding of the molecular mechanisms underlying their anticancer and anti-inflammatory activities remains very limited. Generally, these mechanisms relate to the antioxidative properties of polyphenols, which alter the cell signaling cascades that trigger cell cycle arrest and apoptosis of cancer cells or attenuate their adhesiveness, reducing their metastatic potential (2). Through other mechanisms, polyphenols interfere with cytokine production and immune responses, modulate gut microbiota, or amend gene expression and the pattern of epigenetic marks, such as DNA methylation and histone posttranslational modifications (2). Consumed polyphenols are predominantly polyglycosylated and therefore are characterized by high molecular weight and complex structures, which markedly impede their absorption and penetration of the cell membrane, thus constraining their activity largely to the extracellular space.

Although histones are well recognized as DNA-binding nuclear proteins and major components of chromatin, they are also found on the surface of human blood monocytes, activated lymphocytes, leukocytes, and apoptotic cells (3). In addition, neutrophils release the neutrophil extracellular trap, a web-like complex of chromatin and antimicrobial proteins, into the extracellular space to fight pathogens that are too large for phagocytic uptake (4, 5). Histones are even present in the blood, and their relatively low (∼2 mg/l) concentration can rise dramatically (>200-fold) in conditions such as sepsis, inflammation, or trauma (6). Extracellular and cell surface histones have been shown to stimulate calcium entry and cytokine release, inhibit macrophage phagocytosis, and activate Toll-like receptors to induce hepatic reperfusion and glomerular cell injuries and calcium oscillation (6). Furthermore, the N-terminal histone tails, cleaved by the neutrophil elastase in neutrophil extracellular traps, function as secondary messengers, adding yet another layer of complexity in the immunological response and immune defense mechanisms (4, 5).

Histones do not possess transmembrane or evident membrane-binding domains but can associate with the cell surface through electrostatic interactions with highly negatively charged heparin sulfate proteoglycans or with the negatively charged phosphoserine during phosphoserine exposure to the outer leaflet of the plasma membrane in apoptotic monocytoid cells (3, 7). The Uchida lab has shown that cell surface histones function as receptors for AGE (advanced glycation end products) and regulate macrophage recruitment to the site of inflammation (8). In their new study published in a recent issue of JBC, the Uchida lab reports on characterization of the mechanism by which polyphenols can modify nonhistone proteins and interact with extracellular histones (9).

Yamaguchi *et al.* (9) demonstrate that proteins abundant in the blood, such as albumin, transferrin, and immunoglobulin, once modified by polyphenols, acquire the ability to bind histones. Not all polyphenols help in the developing of this ability; of 25 polyphenols tested, only seven, including (-)-epigallocatechin-3-O-gallate (EGCG), piceatannol, and baicalein, can do so. The authors further show that binding of the proteins modified by polyphenols to histones requires the N-terminal tails of histones, which are enriched in lysine and arginine residues and therefore are highly positively charged. Acetylation of the histone N-terminal tails eliminates this binding, indicating that unprotected (nonacetylated) lysine residues play a role in the interaction.

What is the mechanism underlying these transformations? It might involve the formation of dehydrolysinonorleucine, a lysine-derived crosslink that was previously reported for piceatannol-mediated protein polymerization (10) (shown in Fig. 1 in ref. (9)). In the presence of oxidizing agents, many polyphenols undergo rapid oxidation into quinones, which can form the Schiff base compounds through interacting with the side-chain amino group of lysine. These compounds are further converted into a highly reactive aldehyde that reacts with the side chain amino group of another lysine to produce again a Schiff base but now connecting two lysines *via* their side chains. The resulting “two-lysine crosslink” couples two proteins or two parts of a protein. Indeed, formation of the reactive aldehyde from a lysine analog upon treatment with EGCG and its acquired histone-binding activity implies that the oxidized lysine pathway has a role in binding to histones (9). However, the “two-lysine crosslink” was undetectable in the reaction mixture and suggested an alternative mechanism.

EGCG is known to quickly oxidize and form heterogeneous polymeric structures. The authors propose that EGCG itself could serve as a linker between the tested proteins (albumin) and histones, providing a hub for the formation of large histone-bound aggregates (9). The reversible nature of the binding of histones to the EGCG–protein aggregates excludes the possibility of oxidation of histone lysines and supports noncovalent interactions, which can modulate extracellular activities of both histones and polyphenol-modified nonhistone proteins.

The study by Yamaguchi *et al.* (9) shows an exciting example of a complex action of plant-based antioxidants on extracellular proteins and advances our knowledge of mechanistic aspects of the polyvalent interactions involving dietary polyphenols. These findings not only shed light on the role of polyphenols in attenuating the endothelial toxicity of histones but also highlight topics for future research regarding the oligomeric state of polyphenols and the reason as to why histones are incapable of forming stable Schiff base crosslinks. It will be exciting to see future work addressing differences in action of the structurally diverse subclasses of polyphenols that are consumed by humans with common food, including polyphenols described in the current study: EGCG, which is found in tea, fruit, and chocolate and belongs to catechins; piceatannol, present in high concentration in red wine, grapes, and peanuts and belongs to stilbenes; and baicalein, an herb-derived flavone.

## Conflict of interest

The authors declare that they have no conflicts of interest with the contents of this article.
